# Case Report: Seronegative rhombencephalitis presenting with ocular flutter and ataxia as a potential sentinel manifestation of gastric adenocarcinoma

**DOI:** 10.3389/fimmu.2026.1844323

**Published:** 2026-10-02

**Authors:** Moyuan Quan, Lanxue Meng, Tengfei Sun, Yixuan Zhang, Xueqin Song

**Affiliations:** 1 Department of Neurology, The Second Hospital of Hebei Medical University, Shijiazhuang, Hebei, China; 2 The Key Laboratory of Neurology (Hebei Medical University), Ministry of Education, Shijiazhuang, Hebei, China; 3 Key Laboratory of Neurology of Hebei Province, Shijiazhuang, Hebei, China; 4 Hebei Medical University, Shijiazhuang, Hebei, China

**Keywords:** ataxia, autoimmune rhombencephalitis, gastric adenocarcinoma, ocular flutter, paraneoplastic neurological syndrome

## Abstract

**Objectives:**

This case report aims to highlight the rare clinical phenotype of seronegative autoimmune rhombencephalitis presenting with ocular flutter and ataxia, and to emphasize its potential association with underlying paraneoplastic neurological syndromes.

**Methods:**

The patient was identified through comprehensive clinical neurological evaluation, video-assisted oculomotor analysis, and diagnostic testing including brain MRI, cerebrospinal fluid (CSF) analysis, and an extensive screening of serum/CSF autoantibodies using cell-based assays (CBAs) and immunoblot assay. To rule out other etiologies, extensive infectious panels were assessed. A tissue-based assay (TBA) was employed to identify potential autoimmune reactivity when standard antibody panels were negative. Long-term follow-up was conducted after the patient was discharged.

**Results:**

A 60-year-old male presented with persistent ocular flutter, mild head-neck-tremor, and cerebellar ataxia. While standard autoantibody panels were negative, TBA demonstrated weak neuronal IgG reactivity in serum, whereas the corresponding CSF TBA was negative. The patient demonstrated a favorable response to immunotherapy (intravenous methylprednisolone and IVIG), with near-complete resolution of symptoms. Notably, gastric adenocarcinoma was diagnosed ten months after the onset of neurological symptoms following the emergence of new gastrointestinal symptoms.

**Discussion:**

This case highlights an exceedingly rare presentation of autoimmune rhombencephalitis characterized by ocular flutter as the sentinel symptom. Although no characterized neural autoantibody was identified, weak serum neuronal reactivity on TBA, mild CSF pleocytosis, the clinical phenotype, and the response to immunotherapy supported a suspected immune-mediated process. The subsequent diagnosis of gastric adenocarcinoma ten months later raised the possibility of a paraneoplastic association, although causality could not be established. Our findings underscore that ocular flutter-ataxia can be a diagnostic clue for underlying malignancy; therefore, clinicians should maintain high vigilance and perform longitudinal tumor screening in such patients, even when initial evaluations are negative.

## Introduction

Autoimmune rhombencephalitis is a clinically heterogeneous and diagnostically challenging condition, particularly in seronegative cases where specific biomarkers are lacking. Ocular flutter, a rare oculomotor abnormality, suggests dysfunction of the inhibitory control network in the brainstem or cerebellum and has been associated with autoimmune etiologies (2). This inhibitory control depends on coordinated pontine and cerebellar circuits, including omnipause–burst neuron interactions and modulation by the oculomotor vermis and fastigial nucleus. However, its presentation as the predominant feature of rhombencephalitis is rarely reported.

Paraneoplastic neurological syndromes (PNS) may precede the clinical detection of an underlying malignancy, with neurological manifestations occurring before cancer diagnosis in approximately two-thirds of cases (3). Although PNS has been associated with various solid tumors, gastric adenocarcinoma represents an uncommon tumor association. Here, we report a case of seronegative autoimmune rhombencephalitis presenting with ocular flutter as the initial manifestation, in which the patient was diagnosed with gastric adenocarcinoma ten months later. This case highlights ocular flutter as a potential sentinel manifestation of an otherwise unexplained immune-mediated brainstem–cerebellar syndrome and underscores the importance of considering an underlying malignancy during longitudinal follow-up.

## Case description

A 60-year-old male presented with a one-month history of progressive blurred vision and gait instability, accompanied by dizziness. Frequent abnormal ocular movements (dozens of episodes per minute) were observed (**Supplementary Video 1**). The patient denied diplopia, limb weakness, or febrile illness since onset. His medical history was otherwise unremarkable. He had no history of smoking, alcohol abuse, or exposure to neurotoxins. His family history was negative for autoimmune, neurological, or neoplastic diseases.

Upon admission, systemic examination and vital signs were normal. Neurological evaluation revealed equal, round, and reactive pupils with full extraocular range of motion. Fundoscopic examination was unremarkable. Continuous ocular flutter was present, accompanied by a mild head-and-neck tremor characterized by rhythmic, small-amplitude, horizontal (“no-no”) oscillations without cervical dystonia or abnormal head posturing. While limb strength was preserved, the patient exhibited a broad-based gait and was unsteady during stance with both eyes open and closed, consistent with cerebellar rather than sensory ataxia. Deep tendon reflexes were symmetric (2+) throughout, and sensory examination to pinprick and vibration was intact. The Babinski sign was absent bilaterally.

### Diagnostic assessment and differential diagnosis

Routine peripheral blood tests were unremarkable. Peripheral blood lymphocyte count showed that both the absolute value (915/μl, reference range 90–560/μl) and percentage (31.38%, reference range 5%–18%) of B lymphocytes were above the normal range, while the absolute values and percentages of T cells and NK cells were within normal limits. Cerebrospinal fluid (CSF) analysis revealed normal opening pressure and mildly elevated white blood cell count (12×10^6^/L), with normal protein, glucose, and chloride levels. CSF oligoclonal bands and the IgG index were not assessed during the initial diagnostic evaluation. To rule out other potential causes, comprehensive evaluations were conducted. An infectious etiology was considered less likely based on negative CSF mNGS, the absence of an infectious prodrome, and the overall CSF profile. Toxic and metabolic disturbances were ruled out by normal results on comprehensive metabolic panels and thyroid function tests. Furthermore, a high-resolution brain MRI with and without contrast enhancement showed no evidence of acute infarction, demyelination, or structural lesions in the brainstem or cerebellum (**Figures 1A, B**). At the time of initial presentation, routine serum tumor markers (including CEA, CA19-9, and AFP) were within normal limits, and the patient lacked systemic symptoms such as weight loss or gastrointestinal complaints. Consequently, invasive malignancy screening, such as endoscopy or whole-body PET-CT, was deferred.

**Figure 1:**
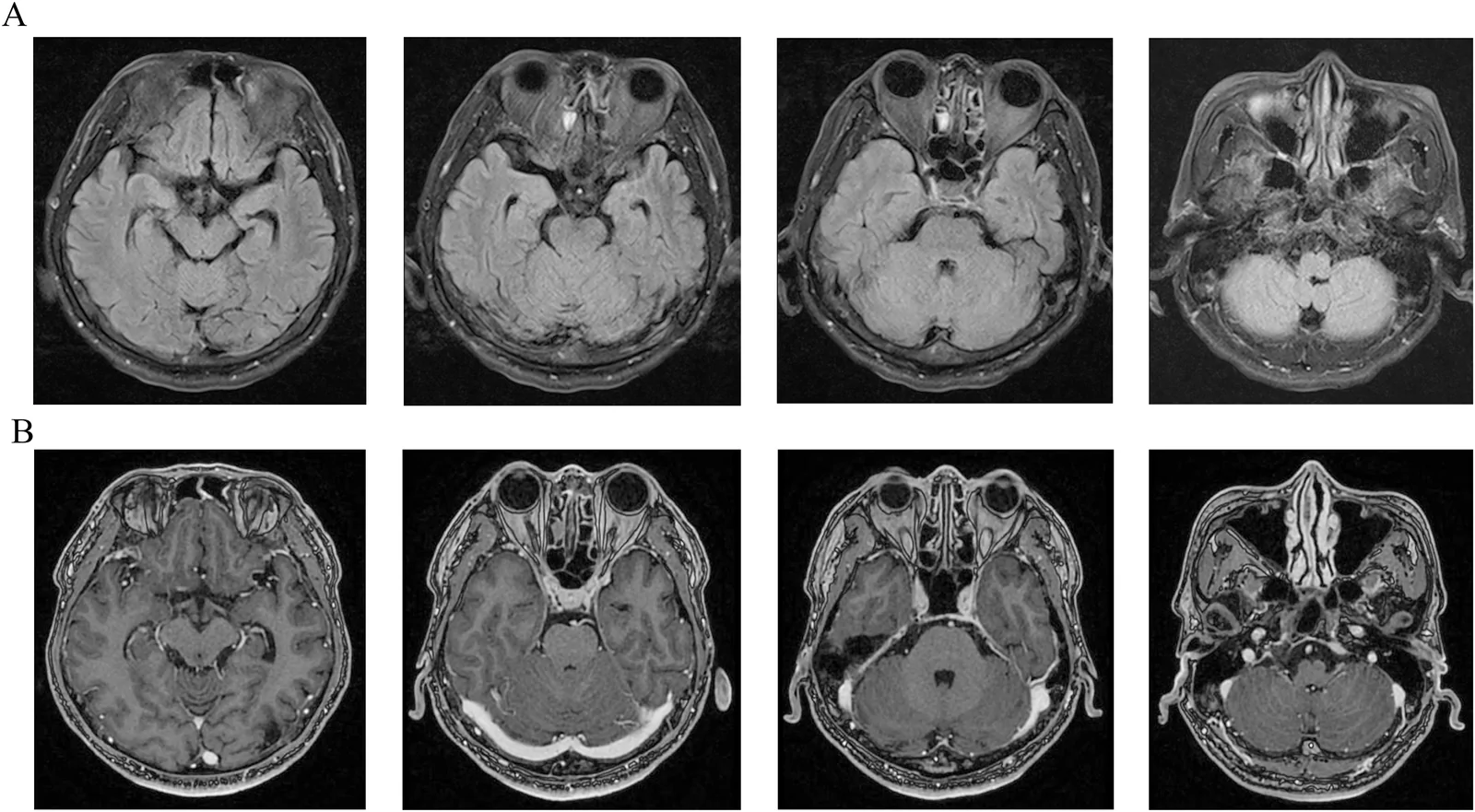
Initial brain magnetic resonance imaging (MRI) of the patient. Axial brain MRI images showing no evident focal signal abnormalities, structural lesions, or abnormal contrast enhancement in the brainstem or cerebellum. **(A)** Axial fluid-attenuated inversion recovery (FLAIR) sequences. **(B)** Axial T1-weighted contrast-enhanced images.

Commercial fixed cell-based assays (CBAs) and immunoblot assay were performed for established neural autoantibodies, including NMDAR, LGI1, CASPR2, GABA-B receptor, IgLON5, mGluR5, AQP4, MOG, Hu, Yo, Ri, Ma2, CV2, amphiphysin and GQ1B, GT1a, GM1-4. All specific antibody tests were negative. The complete antibody panel is provided in **Supplementary Material 1**. Extensive serum and CSF autoantibody panels (**Supplementary Material 1**) were all negative, except for a positive tissue-based assay (TBA) showing characteristic immunofluorescence patterns in serum (**Figure 2**); however, the corresponding CSF TBA was negative (**Figure 3**). Based on the clinical triad, the exclusion of other etiologies, and TBA positivity, a diagnosis of seronegative autoimmune rhombencephalitis was pursued.

**Figure 2:**
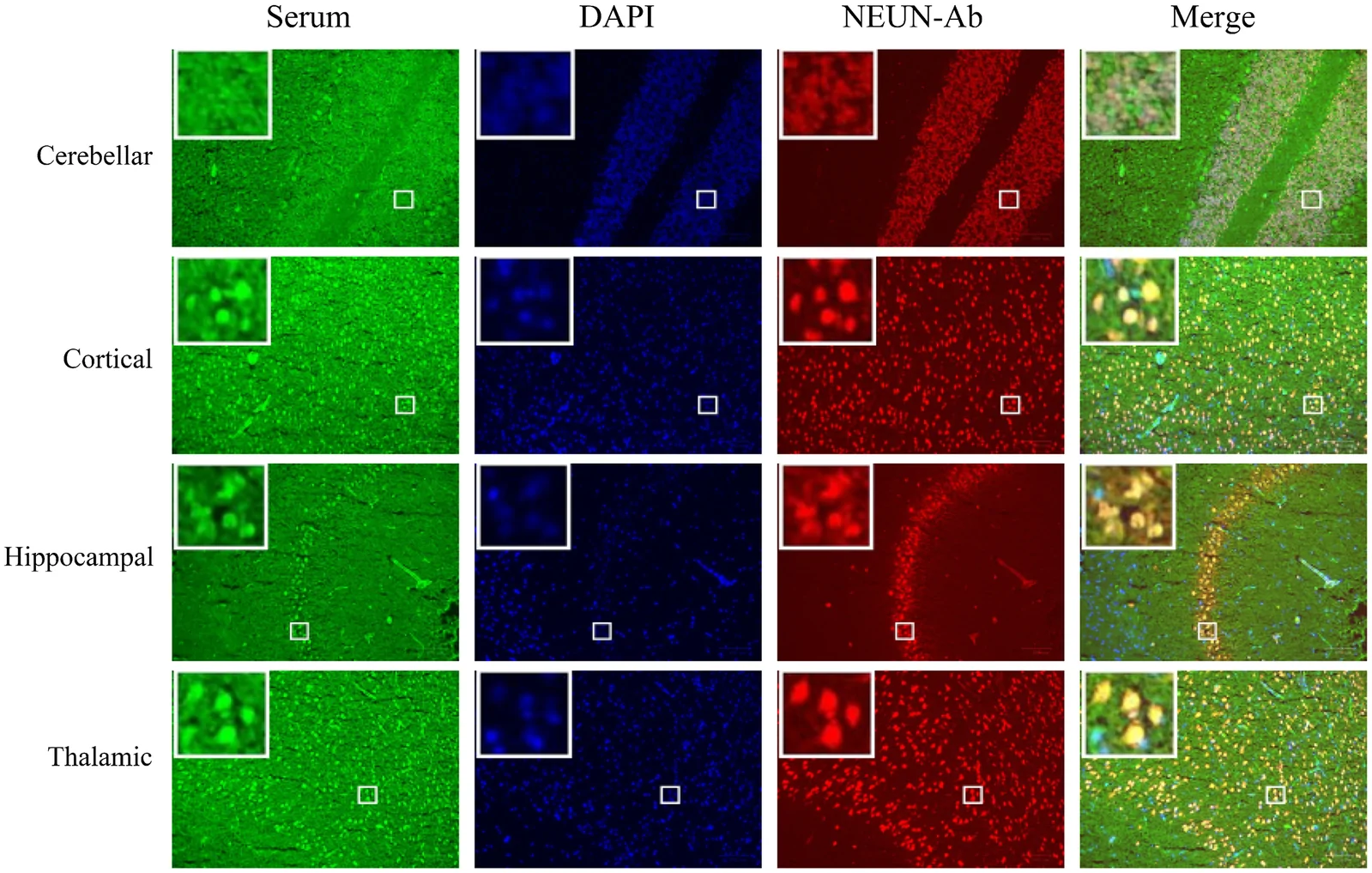
Tissue-based assay (TBA) and co-immunostaining of patient serum. Indirect immunofluorescence assay on rat brain tissue sections showing reactivity of patient serum (green), counterstained with DAPI (blue, for cell nuclei) and a commercial anti-NeuN antibody (red, for neuronal nuclei). Rows from top to bottom show representative brain regions: cerebellum, cerebral cortex, hippocampus, and thalamus. Scattered cellular immunoreactivity was observed across all brain regions in the serum sample (green). Merged images (fourth column) demonstrate colocalization of serum IgG reactivity with NeuN-positive neurons, confirming the presence of anti-neuronal antibodies in the serum.

**Figure 3:**
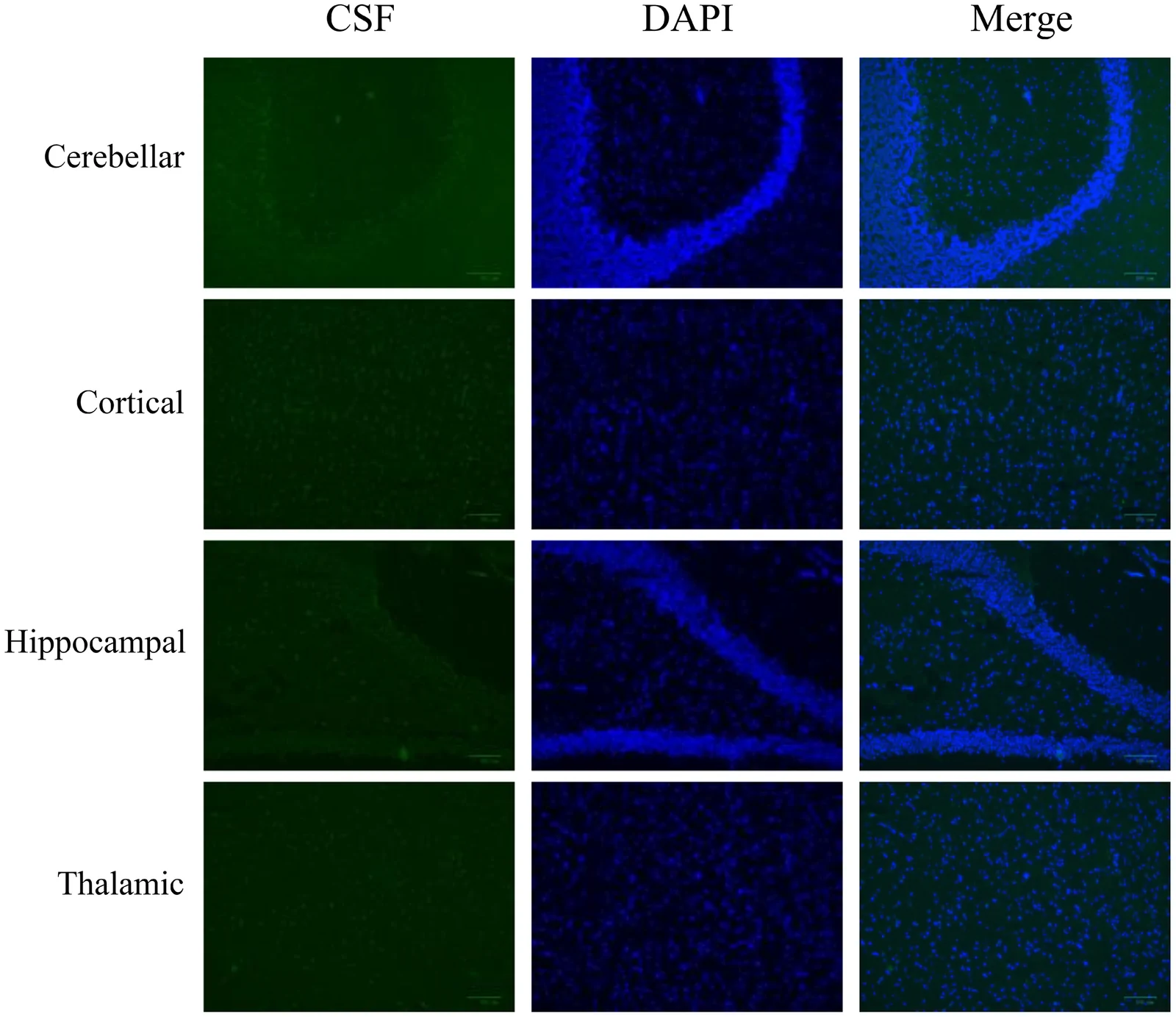
Tissue-based assay (TBA) of patient cerebrospinal fluid (CSF). Indirect immunofluorescence assay on rat brain tissue sections using patient CSF (green), counterstained with DAPI (blue, for cell nuclei). Rows from top to bottom show representative brain regions: cerebellum, cerebral cortex, hippocampus, and thalamus. No detectable specific fluorescent signal or reactivity was observed in any of the brain regions tested (first column), indicating a negative TBA result in the CSF sample. Merged images are shown in the third column.

### Tissue-based assay methodology

To further investigate potential paraneoplastic or autoimmune etiologies despite negative commercial multiplex panels, a tissue-based assay (TBA) was performed by V-Medical Laboratory Co., Ltd. Acetone-fixed cryosections (10 μm) of SD rat brain tissues (including cortex, hippocampus, thalamus, and cerebellum) were incubated with patient serum (1:10 in 0.2% Triton X-100/PBST) or undiluted CSF, followed by detection with FITC-conjugated anti-human IgG secondary antibody (1:200; Jackson ImmunoResearch). To confirm neuronal localization, double immunofluorescence was performed using a rabbit anti-NeuN antibody (Proteintech) and Alexa Fluor 594-conjugated secondary antibody, along with DAPI nuclear counterstaining. Fluorescence imaging (EVOS M5000, Thermo Fisher Scientific) demonstrated weak neuronal IgG reactivity in the serum, with immunoreactivity co-localizing with NeuN-positive neurons. In contrast, no specific neuronal immunoreactivity was detected in the corresponding CSF sample. These findings suggested the presence of uncharacterized serum anti-neuronal reactivity.

### Therapeutic intervention and outcomes

The patient received pulse corticosteroids (intravenous methylprednisolone 500 mg/day for 3 days, followed by 250 mg/day for 3 days, and 80 mg/day for 3 days) and intravenous immunoglobulin (IVIg, 0.4 g/kg/day for 5 days) (1). Within nine days, symptoms markedly improved: the ocular flutter resolved, and gait nearly returned to baseline. The treatment was well-tolerated, and no adverse events were observed during the acute phase.

After discharge, the patient was treated with methylprednisolone tablets 48 mg (tapered by 4 mg per week until reaching 28 mg, then tapered by 4 mg every two weeks) and mycophenolate mofetil 0.5 g twice daily. Regular outpatient follow-up showed good adherence to the medication regimen and no recurrence of neurological deficits. However, ten months after the diagnosis of autoimmune rhombencephalitis, the patient presented with a progressively worsening sensation of choking.

Subsequent evaluation revealed gastric adenocarcinoma with lymph-node metastases involving the lesser curvature of the stomach (**Figure 4**). No follow-up brain MRI was obtained after the initial hospitalization. At the 12-month neurological follow-up (2 months after initiation of oncological therapy), the patient remained free of neurological relapse, with sustained baseline oculomotor and cerebellar function (mRS = 0 from a neurological standpoint).

**Figure 4:**
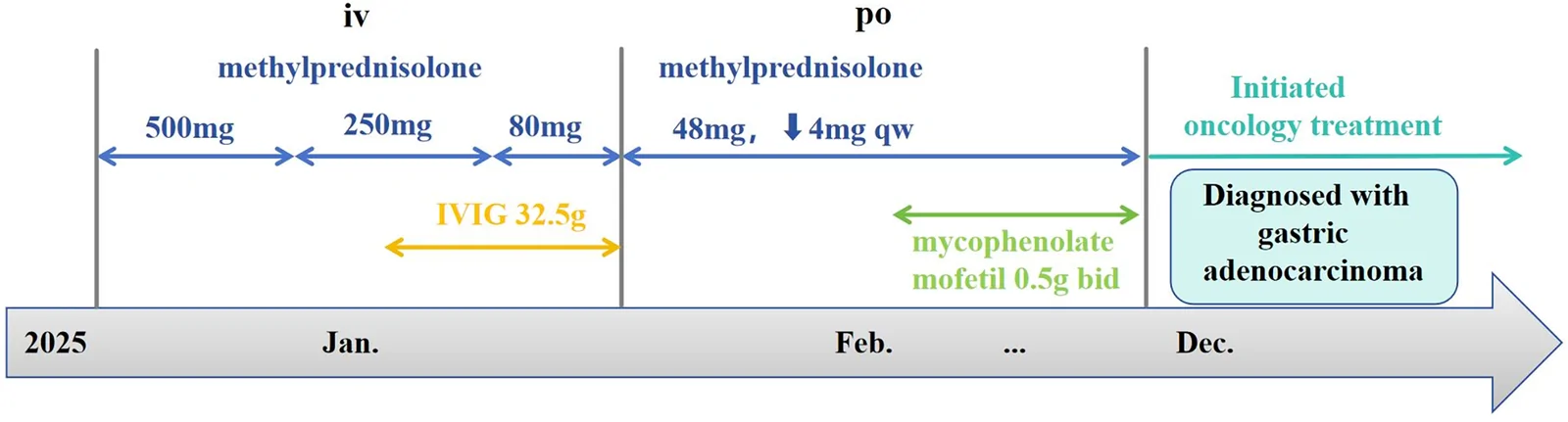
Individualized treatment timeline for the index patient. Treatment was divided into two phases: acute phase and remission phase. In the acute phase, intravenous corticosteroid pulse therapy and adequate-dose IVIG were administered. In the remission phase, maintenance immunosuppressive therapy with low-dose oral corticosteroids and mycophenolate mofetil was given. The patient was diagnosed with gastric adenocarcinoma in December and initiated oncological treatment.

### Patient perspective

The patient reported profound initial distress and anxiety due to the continuous visual disturbances and severe gait instability, which significantly impaired his daily activities. Following the rapid resolution of his neurological symptoms with immunotherapy, he expressed immense relief and gratitude. Although the subsequent diagnosis of gastric adenocarcinoma ten months later was a source of significant emotional distress, the patient appreciated the continuous, long-term clinical follow-up that facilitated subsequent detection of the malignancy, and he was highly motivated to undergo prompt oncological treatment.

## Discussion

We report a case of autoimmune rhombencephalitis presenting with prominent ocular flutter as the initial symptom, with the patient subsequently diagnosed with gastric adenocarcinoma ten months later. The patient was first hospitalized due to blurred vision, and neurological examination revealed marked ocular flutter and mild cerebellar ataxia. Although no characterized pathogenic neural autoantibody or definitive neuroimaging abnormality was identified, weak serum neuronal reactivity on TBA, mild CSF pleocytosis, the clinical phenotype, and the favorable response to immunotherapy supported a suspected immune-mediated brainstem–cerebellar process. Rhombencephalitis presenting with ocular flutter as the initial clinical feature is exceedingly rare, and during follow-up, we proposed the possibility of an underlying paraneoplastic syndrome.

Ocular flutter is characterized by involuntary, high-frequency (typically 10–25 Hz), high-velocity, conjugate saccadic eye movements that oscillate around the visual fixation point. These movements are continuous and notably lack intersaccadic intervals, distinguishing them from other saccadic intrusions or ocular motor oscillations (2, 4). Ocular flutter is thought to reflect instability within an interconnected brainstem-cerebellar saccadic control network (2). At the pontine level, excitatory and inhibitory burst neurons participate in the generation of horizontal saccades, whereas omnipause neurons in the nucleus raphe interpositus provide tonic inhibition of saccadic burst neurons during fixation and transiently cease firing to permit normal saccades (2, 5). At the cerebellar level, the oculomotor vermis and posterior fastigial nucleus contribute to the timing, amplitude, and accuracy of saccades through Purkinje-cell-mediated modulation of cerebellar output (5). Functional interactions between these cerebellar structures and the brainstem saccadic generators are essential for maintaining saccadic stability. Dysfunction of the omnipause-burst neuron system, cerebellar vermis-fastigial circuitry, or their interconnected pathways may impair inhibitory control and permit repetitive back-to-back saccades without normal intersaccadic intervals, thereby providing a plausible neurophysiological basis for ocular flutter (2, 5).

Current reports suggest that adult-onset ocular flutter is associated with infectious (6), paraneoplastic, and autoimmune etiologies (4). In our case, the absence of an infectious prodrome and negative CSF pathogen testing made an infectious etiology less likely, while brain MRI showed no structural or inflammatory abnormalities involving the brainstem or cerebellum. Importantly, a normal brain MRI does not necessarily exclude an immune-mediated brainstem disorder. In a recent cohort of 98 patients with autoimmune brainstem encephalitis, 63% had an abnormal brain MRI, while brainstem T2 hyperintensity was observed in only 31%, indicating that conventional MRI may remain unrevealing in a substantial subset of patients (7). In previously reported cases of rhombencephalitis presenting with ocular flutter, autoantibodies such as anti-GD1b (4), anti-GT1a (4), and anti-GM2 (8) have been detected. Although no characterized neural autoantibody was identified despite extensive testing using cell-based and immunoblot assays, the clinical presentation, mild CSF pleocytosis, weak serum TBA reactivity, and favorable response to immunotherapy supported a suspected immune-mediated process.

The diagnostic assessment of brainstem-predominant autoimmune syndromes requires cautious application of conventional autoimmune encephalitis criteria. The 2016 criteria proposed by Graus et al. provide a syndrome-based framework centered on the subacute development of memory deficits, altered mental status, or psychiatric symptoms together with supportive clinical, CSF, or MRI findings (9). However, subsequent clinical validation demonstrated that patients with isolated brainstem syndromes may not be adequately captured by these criteria (7, 10). Orozco et al. reported that brainstem encephalitis without supratentorial manifestations constituted an important subgroup not fulfilling the conventional criteria for possible autoimmune encephalitis (10). More recently, Gilligan et al. similarly demonstrated that conventional autoimmune encephalitis criteria are not applicable to many patients with autoimmune brainstem encephalitis and proposed brainstem-specific diagnostic criteria (7). Dalmau and Graus further emphasized that, particularly in suspected antibody-negative autoimmune encephalitis, alternative diagnoses should be rigorously excluded and neural antibody testing should combine tissue-based assays with cell-based assays covering a broad range of antigens (11). In the present case, the subacute brainstem-cerebellar syndrome, mild CSF pleocytosis, tissue-based neuronal reactivity, and favorable clinical response to immunotherapy supported a suspected immune-mediated process; nevertheless, the normal MRI and absence of a characterized disease-specific neural antibody warrant cautious interpretation of diagnostic certainty. The therapeutic approach was guided by contemporary best-practice recommendations for suspected autoimmune encephalitis and our institutional protocols for severe immune-mediated neurotoxicity (12). Given the debilitating nature of the brainstem and cerebellar symptoms, prompt escalation to high-dose pulse corticosteroids combined with IVIg was initiated to rapidly halt inflammatory neuronal damage. For the remission phase, the patient was transitioned to a tapering course of oral corticosteroids together with mycophenolate mofetil (MMF) as a steroid-sparing maintenance agent. MMF is among the immunosuppressive agents used for long-term management of autoimmune encephalitis, although evidence supporting maintenance immunosuppression in seronegative disease remains limited and treatment decisions should be individualized (12).

The diagnostic challenge of “seronegative” autoimmune or paraneoplastic rhombencephalitis highlights the intrinsic limitations of commercially available multiplex autoantibody panels. As the landscape of autoimmune testing expands, it is increasingly recognized that apparent seronegativity may merely reflect the presence of novel or rare pathogenic biomarkers not yet included in standard diagnostic kits (13). In this context, TBA serves as a critical complementary tool. While our patient’s samples were processed at a commercial laboratory, we acknowledge that forwarding serum and CSF to specialized national or international neuroimmunology reference laboratories for advanced validation (e.g., live cell-based assays or protein macroarrays) is strongly recommended for atypical cases to identify novel autoantibodies.

Paraneoplastic neurological syndromes (PNS) represent a category of immune-mediated conditions that are closely associated with malignant tumors (14, 15). In a recent cohort of autoimmune brainstem encephalitis, neurological symptoms preceded cancer detection in 69% of patients with an associated malignancy, with a mean interval of 5 months and a reported range of 0–90 months (7). Current PNS-Care recommendations further indicate that the vast majority of associated tumors are identified within 2 years after neurological onset (16), supporting the importance of longitudinal cancer surveillance when the initial screening is negative. These disorders present with a wide range of complex clinical features and may involve both the central and peripheral nervous systems, without being attributed to direct tumor invasion, compression, or metastatic spread. In addition, they cannot be accounted for by complications directly resulting from the tumor itself, as they arise from immune responses triggered by the underlying malignancy. Paraneoplastic brainstem encephalitis/rhombencephalitis is associated with several characteristic tumor types, and the underlying malignancy often varies according to the accompanying neural antibody (7). In a recent cohort of 98 patients with autoimmune brainstem encephalitis, malignancy was identified in 55 patients (56%); the most frequent tumors were testicular germ-cell tumors (n = 20), followed by breast cancer (n = 9) and small-cell carcinoma (n = 6, including four pulmonary cases). Testicular germ-cell tumors were predominantly associated with KLHL11- or Ma2-IgG, whereas small-cell carcinoma was most commonly associated with ANNA-1 (anti-Hu).

In contrast, gastric adenocarcinoma is not among the commonly recognized tumor associations with autoimmune brainstem encephalitis (7, 17). Its detection ten months after neurological onset therefore raises an unusual but unproven paraneoplastic association in the present case. According to the updated 2021 PNS-CARE diagnostic criteria (16), we explicitly calculated the score for this case: Clinical Phenotype (brainstem encephalitis/rhombencephalitis, an intermediate-risk phenotype = 2 points); Antibody Status (seronegative = 0 points); and Oncological Association (gastric adenocarcinoma present but non-classical association = 1 point), resulting in a total score of 3. Thus, the patient did not meet the formal PNS-Care threshold for possible PNS. Nevertheless, the temporal relationship between the neurological syndrome and the subsequent diagnosis of gastric adenocarcinoma raises the possibility of a paraneoplastic association, although a causal relationship cannot be established. This case suggests that ocular flutter in an otherwise unexplained immune-mediated brainstem–cerebellar syndrome may warrant consideration of an underlying malignancy. When clinical suspicion persists despite an initially negative cancer evaluation, individualized longitudinal surveillance may be considered, particularly if new systemic symptoms emerge.

Finally, it is essential to objectively evaluate the clinical management of this case. The strengths of our approach include the longitudinal clinical follow-up and prompt reassessment after the emergence of new gastrointestinal symptoms, the application of TBA to provide supportive evidence for a suspected immune-mediated process, and the rigorous documentation of ocular flutter using video-assisted analysis. However, several limitations must be acknowledged. Primarily, the absence of a defined, specific target autoantibody and the diagnostic reliance on a “weakly positive” TBA result prevented the definitive classification of a specific paraneoplastic antibody at onset. Furthermore, the subsequent detection of gastric adenocarcinoma raises the possibility of a paraneoplastic association; however, the absence of a recognized disease-specific neural antibody makes it difficult to establish a causal relationship between the tumor and the neurological syndrome. A follow-up brain MRI was not obtained, limiting assessment of possible interval radiological changes. In addition, CSF oligoclonal bands and the IgG index were not assessed, which limited the evaluation of intrathecal immunoglobulin synthesis. These limitations highlight the diagnostic complexities of seronegative cases and underscore the necessity for broader application of advanced specialized testing to identify novel autoantibodies in the future.

## Data Availability

The original contributions presented in the study are included in the article/**Supplementary Material**. Further inquiries can be directed to the corresponding author.
