# Supplementary material for: Case Report: Seronegative rhombencephalitis presenting with ocular flutter and ataxia as a potential sentinel manifestation of gastric adenocarcinoma
Source: Front Immunol. 2026 Oct 2;17:1844323. doi: 10.3389/fimmu.2026.1844323 (PMC13672615; doi:10.3389/fimmu.2026.1844323)
Supplement: Supplementary file 1 [file DataSheet1.pdf]

NMDAR (IgG, IgM, IgA), AMPA1/2, LGI1, CASPR2, GABABR, DPPX, IgLON5, GlyRα1, GABAAR (α1, β3, γ2), GABABR2, mGluR1/5/2/8, D2R, Neurexin-3α, GAD65/67, Hu, Yo, Ri, Ma1/2, CV2, Amphiphysin, Tr (DNER), Zic4, PKCγ, Recoverin, Titin, SOX1, KLHL11, AK5, Homer3, ITPR1, ATP1A3, SEZ6L2, NFL, NFH, NFM, ARHGAP26, RAB6a/b, Septin5, GABAARAP, ADAM22/23, GLUR3, MUNC18-1, AP3B2, Syntaxin 1B, SYN1, AChR subunit complex, ANNA3 (DACH1), MAP1B, NCDN, Gephyrin, CA8, TGM2/6, Trib2, TPO, GRIA4, KCTD16, CACNA2D1, PDE10A, GRIK1/2, ROCK2, GRID2, TG, NAE, CACNB1, NBCe1 (SLC4A4), CPT1C, LUZP4, CRMP3/4/2, Neurabin-1, GABACR, AGO, Kv1.1/1.2/1.6, RGS8, SAMD14, Drebrin1, DPP10, INA, PRPH, TRIM9/46/67, BRSK2A, DAGLA, DNM1, VAMP2A, MPO, NSF, PlexinD1, MX1, DBNL, KRT8, KCNA4, AQP1/4, MOG, MBP, MAG, Flotillin1/2, PLP1, GFAP, gangliosides (GM1, GM2, GM3, GM4, GD1a/b, GD2, GD3, GT1a/b, GQ1b, sulfatide), NF155, NF186, CNTN1/2, CASPR1, Gliomedin, NrCAM, Cortactin, LGI4, LRP4, MuSK, RYR1, Agrin, AChR (live CBA), VGCC complex (CACNA1a, CACNB2/3/4), Collagen Q/XIII, TRPC3, UNC5a, DCC, Netrin-1, DOK7, and CHRM1-5.
